# *Rb1* and *Pten* Co-Deletion in Osteoblast Precursor Cells Causes Rapid Lipoma Formation in Mice

**DOI:** 10.1371/journal.pone.0136729

**Published:** 2015-08-28

**Authors:** Emma A. Filtz, Ann Emery, Huarui Lu, Colleen L. Forster, Chris Karasch, Timothy C. Hallstrom

**Affiliations:** 1 Department of Pediatrics, University of Minnesota, Minneapolis, MN, United States of America; 2 Diagnostic and Biological Sciences, School of Dentistry, University of Minnesota, Minneapolis, MN, United States of America; 3 BioNet, Academic Health Center, University of Minnesota, Minneapolis, MN, United States of America; Georgia Regents University, UNITED STATES

## Abstract

The *Rb* and *Pten* tumor suppressor genes are important regulators of bone development and both are frequently mutated in the bone cancer osteosarcoma (OS). To determine if *Rb1* and *Pten* synergize as tumor suppressor genes for osteosarcoma, we co-deleted them in osteoprogenitor cells. Surprisingly, we observed rapid development of adipogenic but not osteosarcoma tumors in the *ΔRb1/Pten* mice. *ΔPten* solo deleted mice also developed lipoma tumors but at a much reduced frequency and later onset than those co-deleted for *Rb1*. *Pten* deletion also led to a marked increase in adipocytes in the bone marrow. To better understand the function of *Pten* in bone development *in vivo*, we conditionally deleted *Pten* in OSX^+^ osteoprogenitor cells using *OSX-Cre* mice. μCT analysis revealed a significant thickening of the calvaria and an increase in trabeculae volume and number in the femur, consistent with increased bone formation in these mice. To determine if *Pten* and *Rb1* deletion actively promotes adipogenic differentiation, we isolated calvarial cells from *Pten* ^*fl/fl*^ and *Pten* ^*fl/fl*^; *Rb1* ^*fl/fl*^ mice, infected them with CRE or GFP expressing adenovirus, treated with differentiation media. We observed slightly increased adipogenic, and osteogenic differentiation in the *ΔPten* cells. Both phenotypes were greatly increased upon *Rb1/Pten* co-deletion. This was accompanied by an increase in expression of genes required for adipogenesis. These data indicate that *Pten* deletion in osteoblast precursors is sufficient to promote frequent adipogenic, but only rare osteogenic tumors. *Rb1* hetero- or homo-zygous co-deletion greatly increases the incidence and the rapidity of onset of adipogenic tumors, again, with only rare osteosarcoma tumors.

## Introduction

Mammalian bone formation occurs in a coordinated, sequential process that is characterized by the commitment of osteoprogenitor cells into pre- and mature osteoblasts whose function is to synthesize the bone matrix that later becomes mineralized. Osteoblasts originate from pluripotent mesenchymal stem cells that can also differentiate into other mesenchymal cell lineages such as adipocytes, chondrocytes, myoblasts, and fibroblasts depending on the nature of the activated transcriptional differentiation program, and if triggered prior to their commitment to the osteoblastic lineage. For example, MyoD transcription factors are critical for the maturation of muscle cell lineages [1], whereas the peroxisome proliferator-activated receptor γ (PPARγ) is necessary for the differentiation of adipocyte lineage cells [2]. Several transcription factors are critical for the commitment of mesenchymal stem cells into the osteoblast cell lineage, such as the *runt*-related genes *Cbfa1* and *Runx2* [3–5]. *SP7*, also known as Osterix and OSX, is a bone-specific transcription factor that regulates osteoblast development and is expressed in precursors at an early stage in the osteoblast lineage [6]. Understanding the regulation of mesenchymal stem cells into distinct lineages is critical because dysregulation or reactivation of these pathways have an important role in the pathogenesis of human disorders such as osteoporosis and osteosarcoma bone tumors.

Osteosarcoma (OS) is the most common primary bone cancer and is associated with excess osteoblastic proliferation. Mutations in several common human cancer genes such as *TP53*, *RB1*, *CDKN2A*, *MYC*, and *PTEN* drive OS development [7, 8]. OS incidence is increased several hundred-fold in patients with *RB1* germline mutations and patients with hereditary retinoblastoma through loss of the Rb tumor suppressor protein, a key regulator of E2F transcription factors and cell cycle progression, and thus its deletion is often accompanied by defects in cell cycle exit and can result in an undifferentiated cellular phenotype [9–11]. In addition, pRB controls mesenchymal cell differentiation through interactions with RUNX2 to promote osteogenic differentiation [12], or through blocking PPAR-γ expression to suppress adipogenesis [13].

*PTEN* (phosphatase and tensin homolog) is a tumor suppressor gene that is mutated at high frequency in a wide variety of human cancers, such as glioblastoma, prostate, breast, osteosarcoma and was recently identified as a key driver of osteosarcoma in a murine forward genetic screen [8, 14, 15]. *PTEN* encodes a lipid phosphatase that dephosphorylates phosphatidylinositol (3,4,5)- triphosphate (PIP3) to oppose the activity of phosphatidylinositol 3-kinase (PI3K), which functions as a PIP3 kinase that is constitutively activated and functions as an oncogene in many cancer types [16]. Loss of *PTEN* causes persistent activation of the Akt serine-threonine kinase which promotes cell growth and proliferation, disables apoptosis, and controls metabolism by directly phosphorylating numerous downstream targets such as BAD, FOXO transcription factors, CASP9, MTOR, MDM2, and others [17].

We recently identified a functional connection between *Rb1* and *Pten* in murine retinal progenitor cells, where *Rb1* deletion deregulates E2F dependent cell cycle entry and cell death, and co-deleting *Pten* inactivates an E2F/FOXO transcriptional complex *in vivo* to prevent apoptosis and promote rapid retinal tumorigenesis in retinal progenitor cells [18]. We tested if co-deleting *Rb1* and *Pten* in mouse osteo-progenitors resulted in osteosarcoma development. We surprisingly observed rapid lipoma formation and increased marrow adipocyte size and numbers in mice with *Rb1* and *Pten* co-deleted in osteoprogenitor cells. Consistent with these observations, deleting *Pten* or co-deleting *Rb1* and *Pten* in calvarial osteoblast cells significantly promoted adipocyte differentiation and adipogenic differentiation gene expression. These data indicate that *Pten* deletion in osteoblast precursors is sufficient to promote frequent adipogenic, but only rare osteogenic tumors. *Rb1* hetero- or homo-zygous co-deletion greatly increases the incidence and the rapidity of onset of adipogenic tumors, again, with only rare osteosarcoma tumors.

## Materials and Methods

### Experimental Animals & Ethics Statement

*Osx1-GFP*::*Cre* mice [6], *Rb1* ^*lox/lox*^ mice [19], *p107* ^*-/-*^ mice [20], and *Pten* ^*lox/lox*^ mice [21] and associated PCR genotyping protocols have been described. Animals were housed in the University of Minnesota animal facility. All mouse experiments were performed in accordance with University of Minnesota Institutional Animal Care and Use Committee procedures and guidelines under IACUC protocols #1309-30948A (2013-present), and previously #0711A20882 (2010–2013). No analgesic or anesthetics were used. Mice were monitored at least three times weekly during the survival study for evidence of surface irritation, infection, inability to obtain feed or water, accompanying weight loss, but these symptoms were not observed. Mice were humanely euthanized with CO_2_ when tumors reached a size of 1cm^3^, or immediately if tumors began to ulcerate the skin, or if a general moribund status evidenced by a reluctance to stand or move around the cage was observed. The University of Minnesota Comparative Pathology Shared Resource assisted with H&E staining, Ki-67 & TUNEL immunohistochemistry (IHC) staining. Kaplan-Meier curves were calculated using GraphPad Prism software, and *p*-values were determined by Student’s t-test. Marrow fat percentage, adipocyte density and size were determined by histomorphometry of mouse tibial sections as described [22, 23].

### Microcomputed tomography (μCT) analysis

Mouse femurs and calvaria were subjected to micro-CT scanning (model XT H 225, Nikon Metrology, Inc.) using the conditions of 90 kV, 90 μA, 0.5° steps with a pixel size at 12 μm and exposure time of 708 ms as described [24]. CT Pro 3D (Nikon metrology) software was used to reconstruct original three-dimensional images. VG Studio Max 2.1 (Volume Graphics GmbH) was used for visualization and three-dimensional rendering and creation of bmp files. Finally, histomorphometry analyses were performed using SkyScan CT-Analyzer Version 1.12 (Bruker micro-CT).

### Calvarial osteoblast isolation, adenovirus infection and cell differentiation

Calvarial osteoblasts were harvested as described [10]. Briefly, calvaria were removed from newborn pups and rinsed in warmed MEM to remove excess tissue. Calvaria were shaken for 20 minutes at 37°C in collagenase digestion mixture and the first fraction was discarded. Calvaria were shaken with another 10 ml aliquot of digestion mix for 40 minutes, and a third aliquot for 90 minutes. Trypsin was quenched with 10% FBS after each digestion, and cells from fractions 2 and 3 were resuspended in 10% FBS MEM and plated on 60mm plates at an approximate density of 1 calvaria/1.5 plates. Adenovirus infections were performed as described [25]. Cells were infected 24 hours after isolation (60–80% confluence) with control, or CRE expressing adenovirus at a multiplicity of infection (MOI) of 100. Adipogenic Media (α-MEM containing 10% FBS, 1% anti-microbial, 0.5 mM 3-isobutyl-1-methylxanthine (IBMX), 5 ug/ml h-insulin, and 0.1 μM dexamethasone) or osteogenic media (α-MEM containing 10% FBS, 1% anti-microbial, 100 mM L-ascorbic acid, 0.01M sodium phosphate) differentiation media was added five days after adenovirus infections. Cells were grown in differentiation media for 25 days, and media was changed every three days.

### Oil Red O (ORO) and Alizarin Red staining of differentiated calvarial cells

ORO stock solution (3mg/mL in isopropanol) was prepared fresh each experiment and mixed at a 3/2 ratio to make the working solution, which was filtered and used within 2 hours. Cells were rinsed with PBS, fixed in 10% formalin for 30 minutes at room temperature, rinsed with water and treated with 60% isopropanol for 5 minutes. ORO working solution was added to cells for 5 minutes and then rinsed with water. Cells that had been treated with osteogenic differentiation media were fixed (10% formalin for 30 minutes). After rinsing fixed cells, alizarin red solution (2% Alizarin Red S in distilled water, pH adjusted to 4.2 with ammonium hydroxide) was added to cells for 15 seconds and then rinsed with water before imaging.

### Quantitative real-time PCR

RNA was prepared from cells for quantitative real-time PCR using RNeasy, QIAshredder, and QuantiTect SYBR Green RT-PCR kits from QIAGEN. Each RT-PCR experiment was performed in triplicate and normalized against expression of GAPDH expression levels and fold changes were calculated using ΔΔCT method [26]. *p*-values were determined by Student’s t–test. *p* < 0.05 were considered significant. Primers are listed in S1 Table.

## Results

### Combined deletion of *Pten* and *Rb1* in osteoblast precursor cells accelerates adipogenic tumor formation

Because the *Rb1* and *Pten* tumor suppressor genes are frequently mutated in bone cancer, we predicted that their combined deletion might provoke a stronger tumor phenotype than individual deletion of either gene. The *Osx-Cre* transgenic mouse was chosen to conditionally delete *Rb1* and *Pten* in these studies because Osterix is expressed in committed osteoprogenitor cells and because *Osx-Cre* has successfully been utilized to generate osteosarcoma mouse models that co-deleted Rb1 and the p53 tumor suppressor genes [6, 27, 28]. We generated *Osx-Cre; Rb1* ^*fl/fl*^; *Pten* ^*fl/fl*^ mice and aged them to determine if they develop OS.

**Fig 1:**
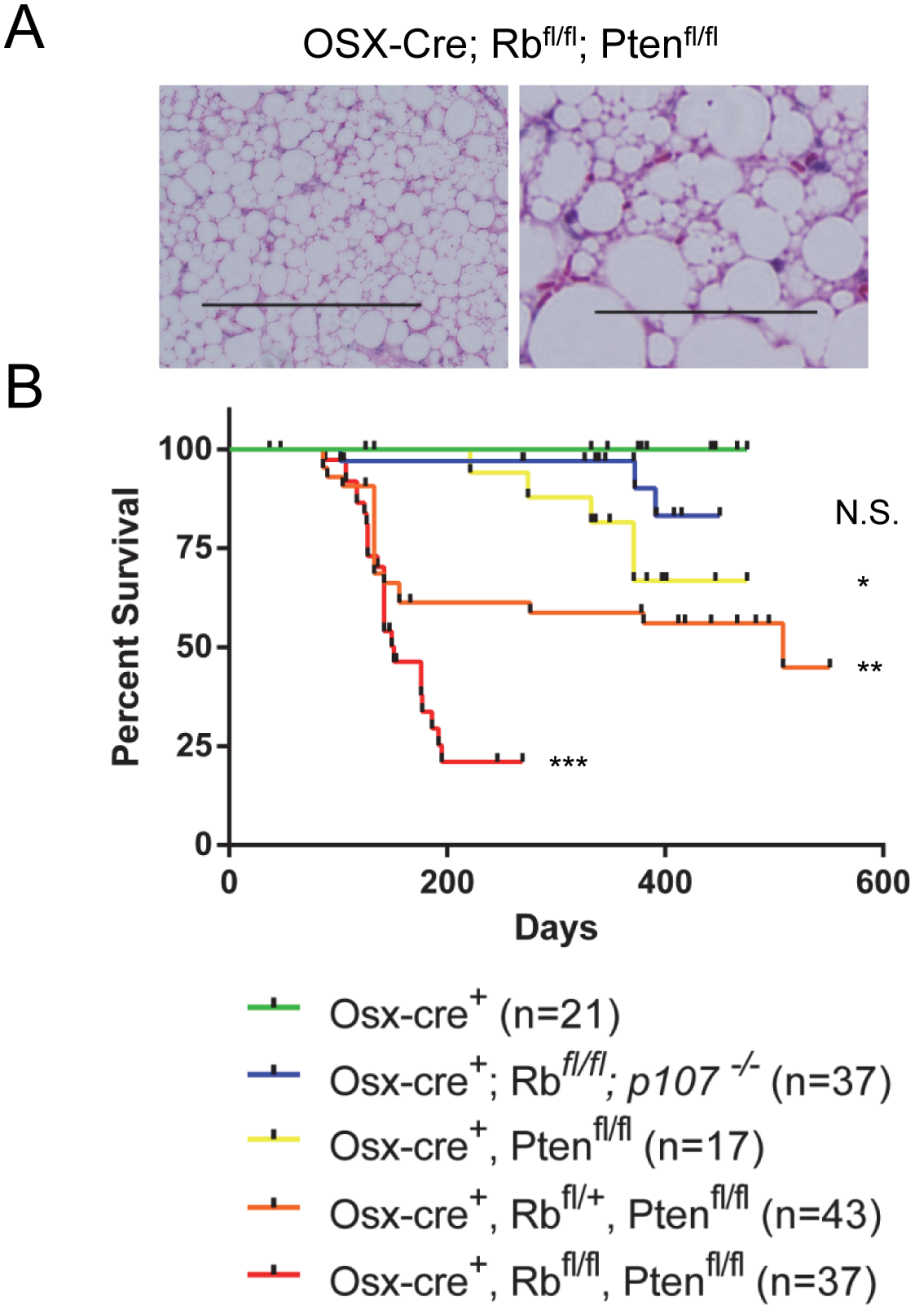
Pten and Rb co-deletion in osteoblast precursor cells causes rapid lipoma but not osteosarcoma tumor onset. (A) H&E staining of sections from lipoma tumors derived from *Osx-Cre; Rb1* ^*fl/fl*^; *Pten* ^*fl/fl*^ mice. Scale bars, 200 μM (Left), 50 μM (Right). (B) Kaplan-Meier survival curve analysis for the indicated genotypes: *Osx-Cre* (n = 21), *Osx-Cre; Rb1* ^*fl/fl*^; *p107* ^*-/-*^ (n = 37), *Osx-Cre; Pten* ^*fl/fl*^ (n = 17), *Osx-Cre; Rb1* ^*fl/+*^; *Pten* ^*fl/fl*^ (n = 43), and *Osx-Cre; Rb1* ^*fl/fl*^; *Pten* ^*fl/fl*^ (n = 37). *p*-values were determined by log rank test comparing a genotype with that of the control mice. *, *p* < 0.05; **, *p* < 0.01; ***, *p* < 0.005; N.S., not significant.

We unexpectedly observed rapid development of large, bilateral adipogenic tumors in 100% (37/37) of the *Osx-Cre; Rb1* ^*fl/fl*^; *Pten* ^*fl/fl*^ mice within the subcutaneous space of the axillae and interscapular/dorsal thoracic region. These unencapsulated, non-invasive masses were detectable before one month of age, and grew rapidly until the tumor burden required mice to be euthanized. Tumors were composed of a multilobulated mass of well-differentiated white and brown fat that was interspersed and supported by multifocal to coalescing thin bands of fibrous connective tissue, stroma, and rare macrophages (Fig 1A). The mass was composed mainly of white fat (large vacuoles with eccentric nuclei) and rare, interspersed brown fat (small multi-vacuolated cells). The surrounding tissues were largely skeletal muscle and brown fat. These animals displayed little subcutaneous, retroperitoneal, or mesenteric fat stores, besides the large tumors. Detailed radiographic and histologic examination of the bones (skull, mandible, thoracic vertebrae and femur) from control and *Osx-Cre; Rb1* ^*fl/fl*^; *Pten* ^*fl/fl*^ mice revealed no bone lesions in any of these animals (data not shown).

Because the RB protein is a reported positive regulator of osteogenic differentiation, we predicted that retaining one copy of the *Rb1* gene in the *Osx-Cre Rb1* ^*fl/+*^; *Pten* ^*fl/fl*^ mice might shift the tumor formation away from adipogenic and towards osteogenic tumors. Cohorts of control, *Osx-Cre; Pten* ^*fl/fl*^, and *Osx-Cre; Rb1* ^*fl/+*^; * Pten* ^*fl/fl*^ mice were generated and aged to around 18 months to assess tumor incidence. We also generated *Osx-Cre; Rb1* ^*fl/fl*^; *p107* ^*-/-*^ mice to test the effect of co-deleting the *Rbl1* (p107) gene, a pRB pocket protein family member, because its deletion augments other Rb driven tumors like retinoblastoma in mice [29]. However, we found that the *Osx-Cre Rb1* ^*fl/+*^; *Pten* ^*fl/fl*^ mice still developed adipogenic tumors, albeit at a lower incidence and reduced rapidity compared to those arising in the *Osx-Cre; Rb1* ^*fl/fl*^; *Pten* ^*fl/fl*^ mice (Fig 1B). Retention of both copies of *Rb1* (*Osx-Cre; Pten* ^*fl/fl*^) reduced the formation and delayed onset of lipoma formation even further, and only 4 of 17 mice developed them at around 9–12 months old. We detected a single sternal OS on the *Osx-Cre; Pten* ^*fl/fl*^ (1/7 mice analyzed) and a single OS, also sternal, on one of the *Osx-Cre Rb1* ^*fl/+*^; *Pten* ^*fl/fl*^ mice (S1 Fig). No detectable OS or adipogenic tumors or other abnormal phenotype were detected on the control or *Osx-Cre; Rb1* ^*fl/fl*^; *p107* ^*-/-*^ mice. These data indicate that Pten deletion in osteoblast precursors strongly promotes lipoma tumor formation, that *Rb1* hetero- or homo-zygous co-deletion greatly increases the incidence and the rapidity of onset of adipogenic tumors, with only a modest increase in detectable osteosarcoma tumors compared to control mice.

### *Pten* loss in osteoprogenitor cells leads to accumulation of bone marrow adipocytes

**Fig 2:**
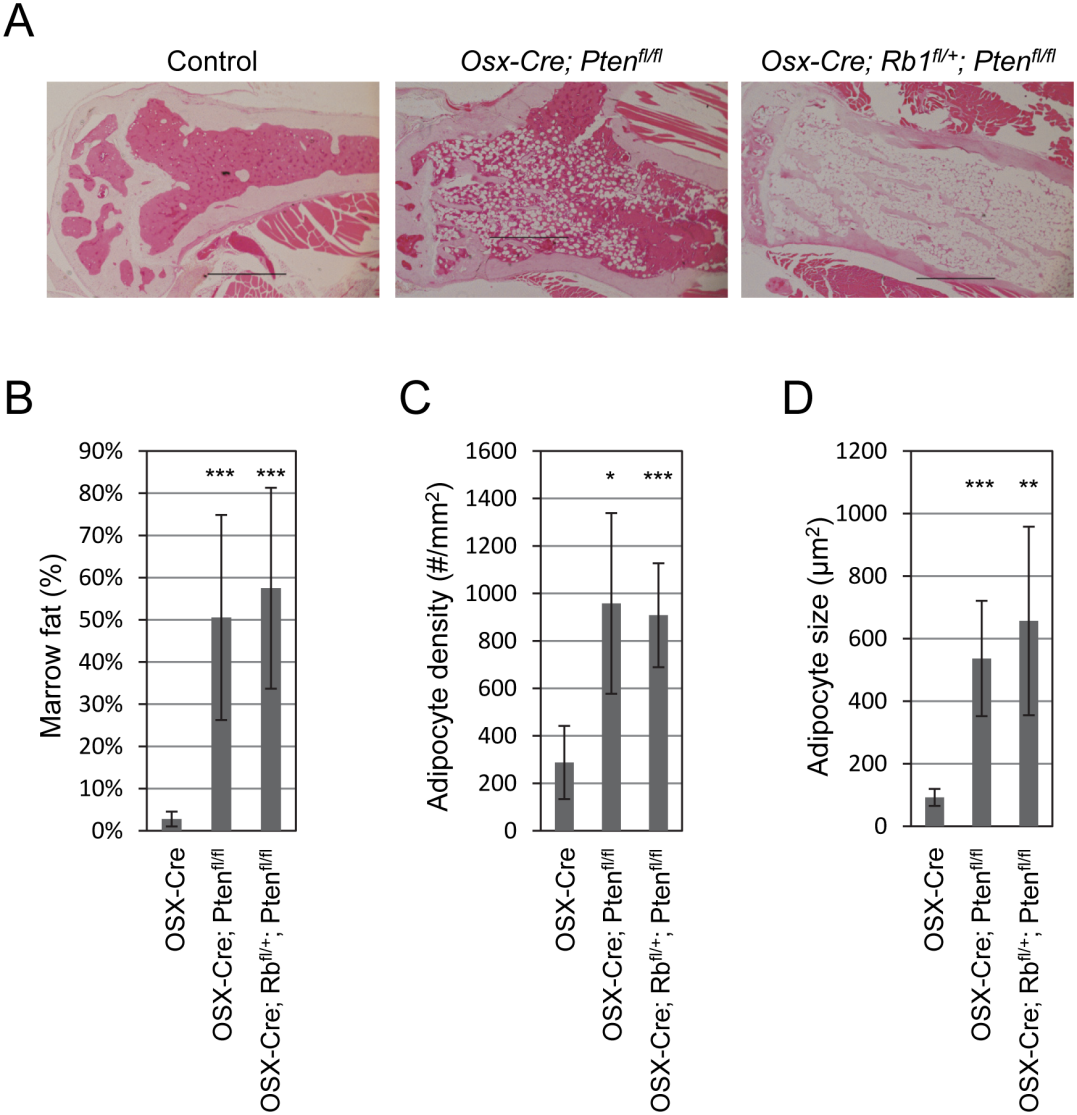
Pten disruption in osteo-progenitor cells increases marrow adipocyte density and size. (A) Tibias from 12-month old *Osx-Cre* (control), *Osx-Cre; Pten* ^*fl/fl*^, and *Osx-Cre; Rb1* ^*fl/+*^; *Pten* ^*fl/fl*^, were cut for H&E staining and proximal end displayed. Scale bars, 500 μM. Tibial marrow contents from 12-month old mice were compared using NIH ImageJ. (B) Percentage of marrow fat; (C) adipocyte number (#/mm^2^); (D) adipocyte size (μm^2^) were measured using histomorphometry. Data are the mean ± SD from six mice. *, *p* < 0.05; **, *p* < 0.01; ***, *p* < 0.005.

*Osx-Cre* expresses Cre in several mesenchymal cell types in the bone marrow of post-natal mice, including osteoblasts, osteocytes, chondrocytes, adipocytes, and stromal cell [30]. Thus, it is not entirely clear if *Osx-Cre* targets a common MSC progenitor of these cell types or if it targets discrete progenitor cell types. We analyzed sections of tibias from 12 month-old control, *Osx-Cre; Pten* ^*fl/fl*^ and *Osx-Cre; Rb1* ^*fl/+*^; *Pten* ^*fl/fl*^ mice by hematoxylin and eosin (H&E) staining to determine the consequences of homozygous deletion of *Pten* with, or without, heterozygous deletion of *Rb1*. We observed a striking increase in the number of adipocytes in the proximal tibial sections of *Osx-Cre; Pten* ^*fl/fl*^ and *Osx-Cre; Rb1* ^*fl/+*^; *Pten* ^*fl/fl*^ compared with control mice (Fig 2A). We used histomorphometry to quantify the differences in marrow adipocytes between the three strains. Total marrow fat increased from 3% in control mice to over 50% in *Osx-Cre; Pten* ^*fl/fl*^ and *Osx-Cre; Rb1* ^*fl/+*^; *Pten* ^*fl/fl*^ mice (Fig 2B). This difference was due to significant increases in adipocyte density (Fig 2C) and average adipocyte size (Fig 2D). These differences were due to *Pten* deletion because additional heterozygous loss of *Rb1* did not augment the phenotype.

### *Pten* deletion in mouse osteoblast precursors results in increased calvaria thickness and bone volume

**Fig 3:**
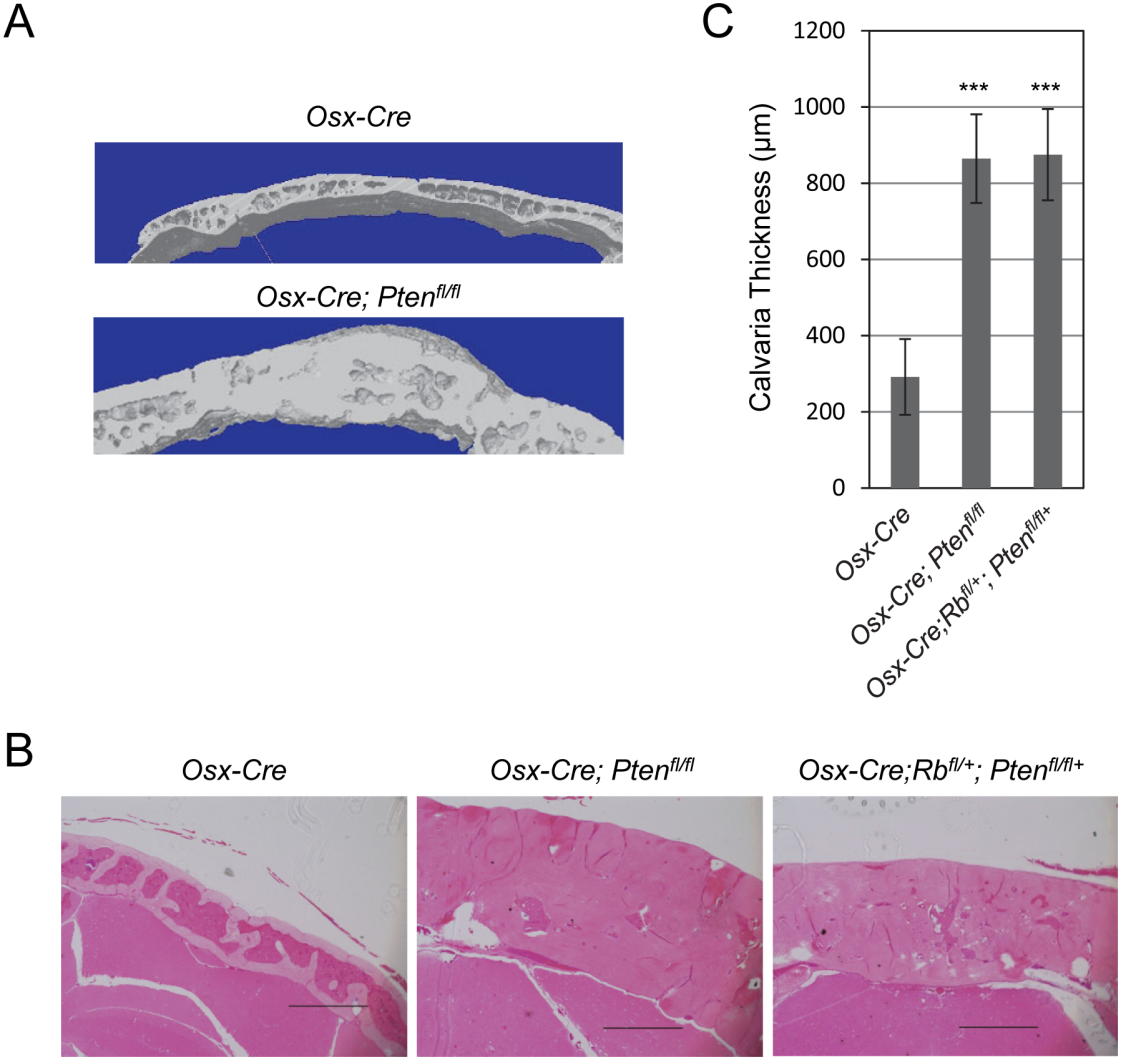
Disruption of the *Pten* gene in osteoblast precursors leads to increased calvaria thickness. (A) μCT analysis was performed on 12-month-old *Osx-Cre* (control) and *Osx-Cre; Pten* ^*fl/fl*^ calvaria. (B) Representative coronal calvarial sections from 12-month old mice. Scale bar, 500 μM. (C) Bar graphs showing the calvarial thickness (mean ± SD; n = 6). ***, *p* < 0.005.

We evaluated the effects of *Pten* deletion on skeletal anatomy using three-dimensional micro-computed tomography (μCT) analysis and histology. μCT analysis revealed a significant thickening of the calvaria of 12-month-old *Osx-Cre; Pten* ^*fl/fl*^ mice compared to those from control mice (Fig 3A). We also analyzed calvaria by histology from control, *Osx-Cre; Pten* ^*fl/fl*^ and *Osx-Cre; Rb1* ^*fl/+*^; *Pten* ^*fl/fl*^ mice (Fig 3B). Calvaria were on average 3-times thicker in both the *Osx-Cre; Pten* ^*fl/fl*^ and *Osx-Cre; Rb1* ^*fl/+*^; *Pten* ^*fl/fl*^ compared to control mice (Fig 3C).

**Fig 4:**
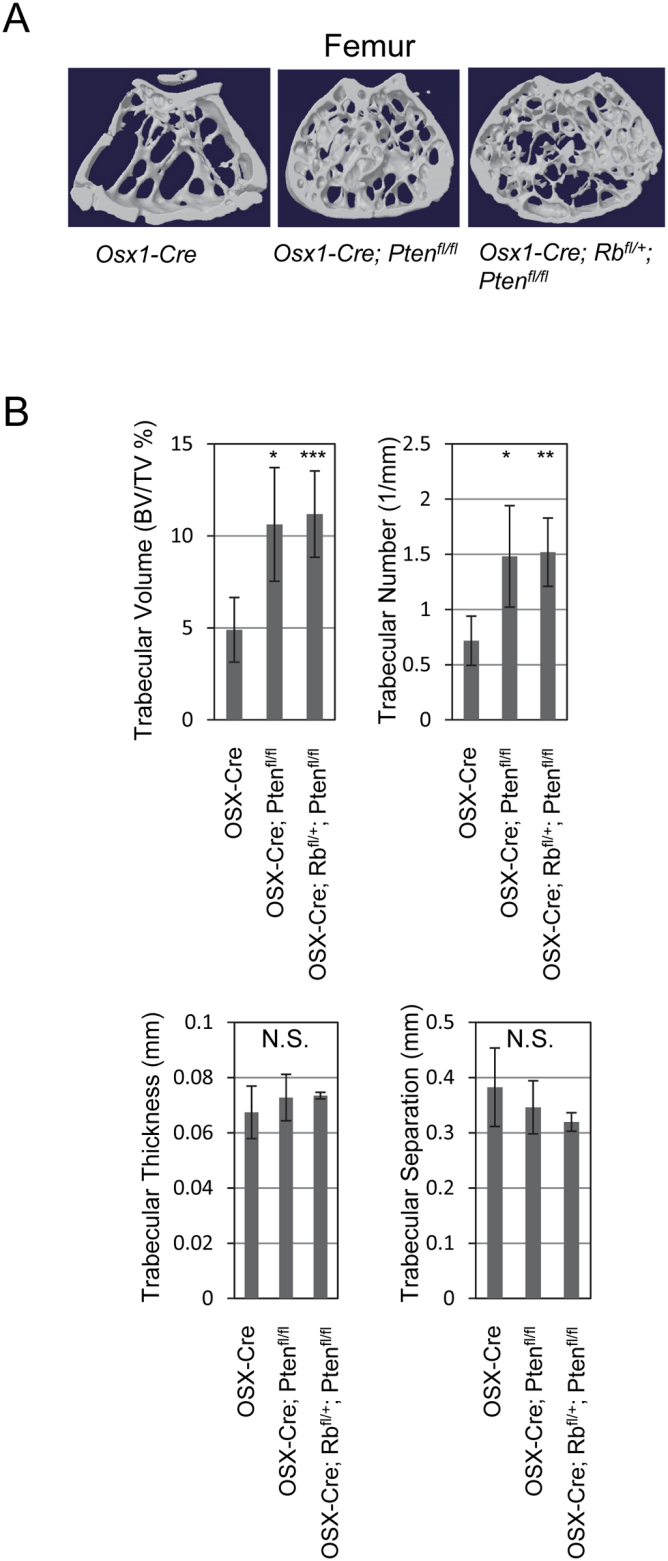
*Pten* deletion in osteoblast precursors increases trabecular bone volume. (A) μCT analysis was performed on the distal femur of control, *Osx-Cre; Pten* ^*fl/fl*^ and *Osx-Cre; Rb1* ^*fl/+*^; *Pten* ^*fl/fl*^ mice. (B) Bar graphs showing the trabecular volume (BV/TV%), trabecular number (1/mm), trabecular thickness (mm), trabecular separation (mm) (mean ± SD; n = 6). *, *p* < 0.05; **, *p* < 0.01; ***, *p* < 0.005; N.S., not significant.

We next analyzed femurs by μCT from control, *Osx-Cre; Pten* ^*fl/fl*^ and *Osx-Cre; Rb1* ^*fl/+*^; *Pten* ^*fl/fl*^ mice. Whereas the cortical thickness of the *Osx-Cre; Pten* ^*fl/fl*^ and *Osx-Cre; Rb1* ^*fl/+*^; *Pten* ^*fl/fl*^ femurs was unchanged compared to control (Fig 4a), both the trabecular volume density and trabecular number were significantly increased in *Osx-Cre; Pten* ^*fl/fl*^ and *Osx-Cre; Rb1* ^*fl/+*^; *Pten* ^*fl/fl*^ mice compared to control animals (Fig 4a and 4b). Bone volume over total volume (BV/TV %) significantly increased from 5% in control mice to 10% in *Osx-Cre; Pten* ^*fl/fl*^ and *Osx-Cre; Rb1* ^*fl/+*^; *Pten* ^*fl/fl*^ 12-month-old mice. Likewise, trabecular number increased from 0.7 (1/mm) to 1.5 (1/mm) at one year in the Pten-deleted and *Osx-Cre; Rb1* ^*fl/+*^; *Pten* ^*fl/fl*^ mice (Fig 4b). Neither trabecular thickness nor trabecular separation showed any significant differences between control and experimental animals. These results indicate that *Pten* deletion in OSX^+^ osteoprogenitor cells results in increased bone mass *in vivo*. Additional heterozygous loss of the *Rb1* tumor suppressor gene (*Osx-Cre; Rb1* ^*fl/+*^; *Pten* ^*fl/fl*^) did not increase any of these parameters compared to *Pten* deletion alone.

### *Rb1* and *Pten* control adipocyte and osteoblast differentiation

**Fig 5:**
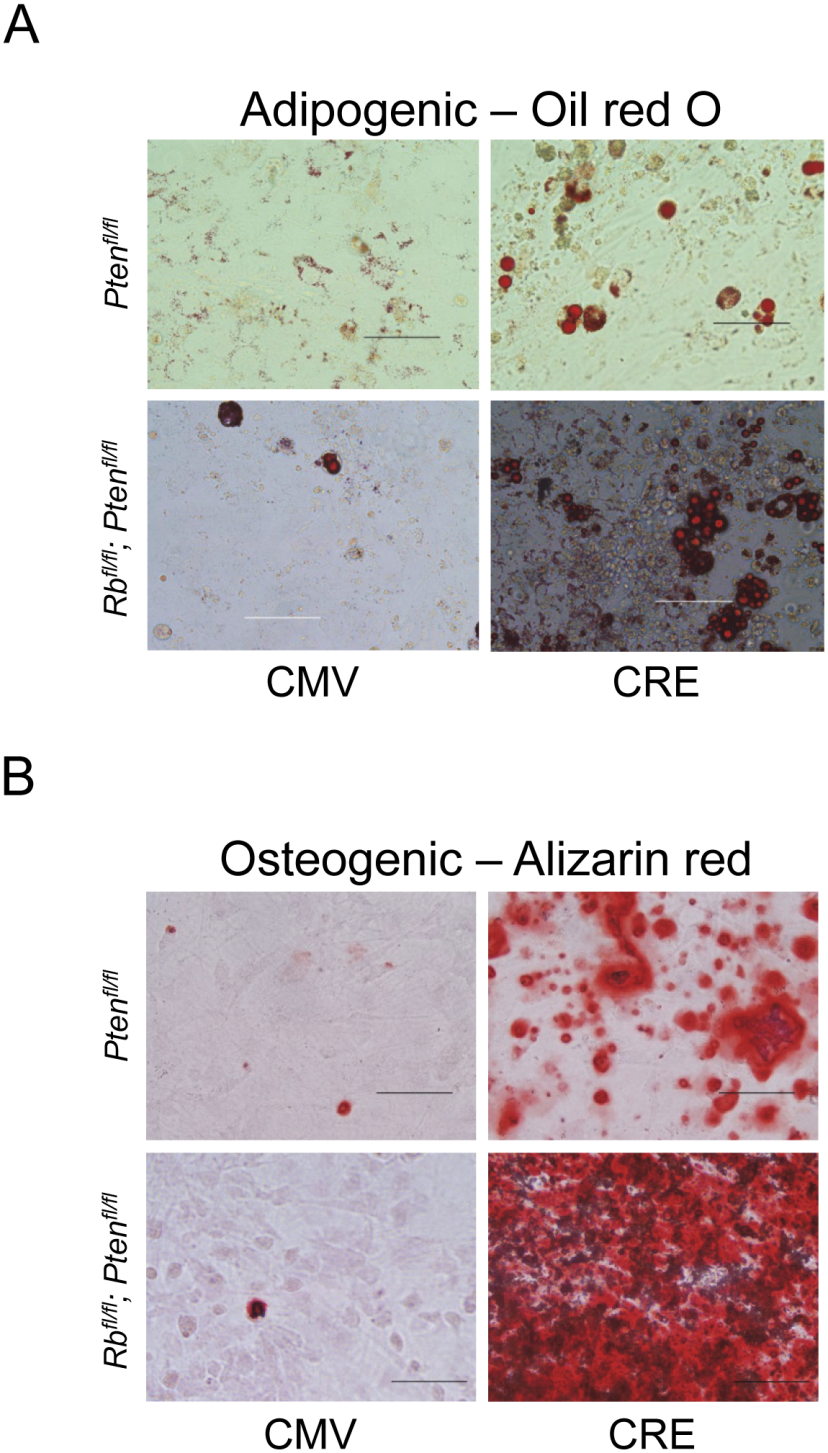
Loss of *Pten* promotes primary calvaria cell differentaion into adipocytes and osteoblasts and is further accentuated by co-deletion of *Rb1*. (A) Primary calvarial osteoblasts were grown to confluence, infected with control or Adeno-Cre, and grown for 25 days with adipogenic differentiation media and measured for adipocyte differentiation by Oil red O staining. Scale bar: 50 μM. (B) Cells were cultured as in (A) but were treated with osteoblastic differentiation media. Terminal osteoblastic differentiation was measured by alizarin red staining. Results are representative of 6 experiments.

Since *Pten* deletion in osteoprogenitor cells caused an increase in adipocytes *in vivo*, it is possible that it directly regulates adipocytic differentiation. We tested this using an established *in vitro* osteoblast differentiation assay. Primary cells were isolated from the calvaria of newborn mice carrying conditional *Pten* ^*fl/fl*^ or *Rb* ^*fl/fl*^ */Pten* ^*fl/fl*^ and grown to confluence. Cells were then infected with control or Cre-recombinase expressing adenovirus, and gene deletion was confirmed by PCR (data not shown). Primary plated cells were treated with standard adipogenic or osteogenic differentiation media for 25 days. Using this approach, cells that differentiate into adipocytes contain lipid droplets that can be detected by staining with Oil red O. Likewise, differentiated osteoblasts secrete calcium deposits which can be detected by staining with alizarin red. Oil red O staining on calvarial cells lacking *Pten* showed increased adipocyte differentiation compared with control cells (Fig 5a). Adipocyte differentiation was more pronounced by co-deleting *Rb1* and *Pten*. Deletion of *Pten* also increased osteoblast differentiation as detected by alizarin red staining (Fig 5b). The *Rb* ^*fl/fl*^ */Pten* ^*fl/fl*^ cells differentiated to a greater extent than *Pten* ^*fl/fl*^ cells by alizarin red staining. These data show that loss of *Pten* acts in an intrinsic manner to increase calvarial cell differentiation into adipocytes and osteoblasts, and that co-deleting *Rb1* with *Pten* augments differentiation into both cell types.

**Fig 6:**
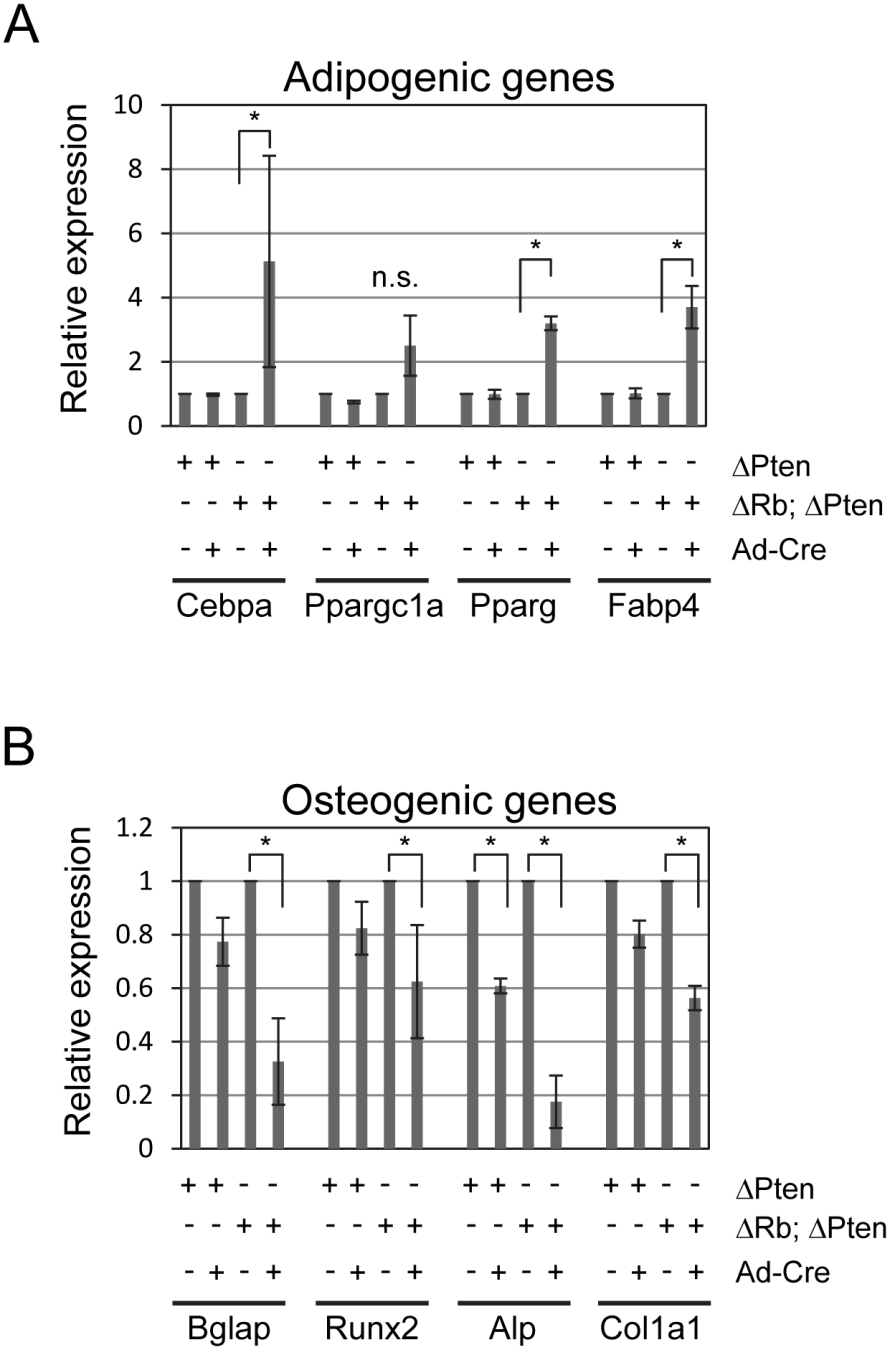
Effects of *Pten* deletion and *Rb1/Pten* co-deletion on adipogenic and osteoblastic gene expression. Calvarial osteoblasts were isolated, cultured, and infected with control or Adeno-Cre and differentiated with adipogenic (A) or osteogenic (B) differentiation media for 48 hours. Quantitative PCR was performed on isolated RNA and displayed as fold-change in relative expression. Data are the mean ± SD from three independent experiments. *, *p* < 0.05.

We then measured changes in expression of several adipogenic and osteogenic genes by qPCR to further examine the differentiation characteristics of these cells. Gene expression was assessed 48 hours after adding adipogenic or osteogenic media to detect early effects on differentiation. Deletion of *Pten* had no effect on any of the pro-adipogenic *Cebpa*, *Ppargc1a*, *Pparg*, or *Fabp4* genes tested at this time point (Fig 6a). Co-deleting *Rb1* with *Pten*, however, significantly induced expression of *Cebpa*, *Pparg*, and *Fabp4*, but not *Ppargc1a*, at this timepoint. *Pten* deletion caused slight, but non-significant decreased expression of the *Bglap*, *Runx2* and *Col1a1* but significantly decreased *Alp* pro-osteoblastic gene expression. *Rb1* and *Pten* co-deletion, however, significantly reduced expression of each gene. These findings suggest that *Pten* deletion had only modest effects on adipogenic and osteogenic gene expression at early timepoints but was capable of promoting both adipogenic and osteogenic differentiation in longer assays. Second, co-deleting both *Rb1* and *Pten* significantly increased adipogenic gene expression, concomitant with increased adipogenesis. It is unclear why *Rb1* and *Pten* co-deletion reduced osteogenic gene expression at early time points but robustly induced osteogenesis in this *in vitro* model.

## Discussion

In this study we deleted *Rb1* and *Pten* from osteoprogenitor cells to determine the consequences for bone development and tumor formation. We show that *Pten* loss significantly increased bone accumulation over time, but also caused a greatly increased adipocyte population and size in the bone marrow. *Pten* deletion also caused lipoma formation outside the marrow, which was robustly accelerated by co-deleting *Rb1*. A variety of other cre-deleter mouse strains have also been used to demonstrate that *Pten* is an important contributor to normal skeletal growth. *Pten* deletion in mature osteoblasts using an *Osteocalcin-cre* driver increased bone cell proliferation and skeletal mass over the lifespan of the animal [31]. Several groups conditionally deleted *Pten* in osteochondroprogenitor cells using *Col2a1-cre* transgenic mice, leading to *Pten* deletion in cartilage and perichondrium. These mice too exhibited skeletal overgrowth, and also chondrocyte dysplasia, and one displayed increased lipoma formation [32–34]. Deletion of *Pten* in mesenchymal cells using *Dermo-cre* caused early lethality in the majority of mutant mice, but surviving mice exhibited increased osteoblast differentiation and bone mass [35, 36]. Thus, *Pten* deletion across all stages of osteoblast formation, from early mesenchyme, partially differentiated progenitors, and mature osteoblasts, consistently increases bone growth *in vivo*. Mechanistically, *Pten* loss may promote bone growth by inactivating the FOXO transcription factors. FOXOs are phosphorylated and inactivated by the AKT serine/threonine kinase, which is constitutively activated following *Pten* loss. *Foxo1*, *-3*, *and -4* co-deletion using *Osx1-Cre* leads to increased osteoblast numbers and high bone mass by increasing β–catenin/TCF mediated transcription, indicating a critical role for FOXOs in suppressing bone formation and osteoblast proliferation [37].

Despite *Pten* deletion strongly promoting osteoblast proliferation and significantly increasing bone mass, and the genomic region containing the *Pten* locus being commonly lost in human and canine osteosarcomas [38, 39], *Pten* deletion in mature osteoblasts or osteoblast precursor cells only rarely leads to osteosarcoma formation. For example, just 2/12 *Col2a1-Cre; Pten* ^*fl/fl*^ mice developed OS [32], and likewise, we observed only one osteosarcoma formation from 17 *Osx1-Cre; Pten* ^*fl/fl*^ mice aged to 18 months. Intriguingly, the *Pten* gene was recovered as one of the key drivers of OS in a murine transposon-based forward genetic screen [8]. Thus, it seems likely that although *Pten* loss is associated with OS progression in various species, *Pten* loss is insufficient to initiate OS without other cooperating mutations. Additionally, complete deletion of *Pten* may favor differentiation towards other lineages, whereas heterozygous deletion of *Pten* may instead favor OS [8].

We observed a striking increase in marrow adipogenesis in the *Pten* deleted mice. *Pten* deletion in the cartilage and perichondrium mesenchymal lineages of *Col2a1-cre* transgenic mice also displayed increased marrow adipocytes and lipoma formation [33]. In contrast, *Pten* deletion in mature osteoblasts using *Osteocalcin-cre* was not reported to affect adipogenesis [31]. *Foxo1*, *-3*, *and -4* co-deletion using the *Osx1-Cre* line leads to decreased marrow adiposity, in contrast with our findings that *ΔPten* increases marrow adiposity, indicating that *Pten* deletion regulates adipose levels in the marrow through FOXO independent targets [37]. *Pten* deletion also promotes adipogenesis in other tissues and cell types. For example, hepatocyte-specific deletion of *Pten* in mice promotes accumulation of adipocytes [40]. Likewise, Pten deletion in the endometrium by *Amhr2-Cre* results in accumulation of adipocytes in the myometrium [41]. This pro-adipogenic effect is tissue specific, and is not observed when *Pten* is deleted from skeletal muscles or cardiomyocytes [42, 43]. Deletion of *Pten* in adipocytes leads to striking lipoma formation when targeting MYF5 expressing cells, but surprisingly did not affect lipid mass when deleted from mature *aP2-cre* targeted fat cells [44, 45]. The effects of *Pten* deletion on adipogenesis and fat production appear to depend on the timing of *Pten* deletion and potentially also of the capacity of the targeted cells to trans- or de-differentiate into adipocytes.

Although the *Rb1* gene is also frequently mutated in human osteosarcoma [46], previous studies indicated that *Rb1* deletion in osteoblast precursors only has modest effects and does not promote OS [27, 28]. In general, *Rb1* promotes osteogenesis through interactions with the RUNX2 transcription factor, and inhibits adipogenesis by repression expression of PPARγ[12, 13]. Accordingly, *Rb1* deletion in the embryo using *Meox1-Cre* reduces bone formation and osteogenic gene expression, and simultaneously increases expression of adipogenic markers and adipogenesis [11]. The ability of pRB to suppress adipogenesis has also been observed in the context of mesenchymal p53 deletion. p53 is a major osteosarcoma tumor suppressor, and its homozygous deletion in early mesenchymal precursors (*Prx1-cre*) or partially differentiated osteoblasts (*Osx1-cre*) leads to robust osteosarcoma emergence [11, 27, 28]. Consistent with a role for *Rb1* in suppressing adipogenesis, homozygous *Rb1* co-deletion dramatically shifted the tumor spectrum towards fatty tumors and reduced incidence of OS. Surprisingly, retaining a single copy of *Rb1* in p53 deleted cells shifted the tumor spectrum strongly back towards osteosarcomas, consistent with the notion that *Rb1* is a positive regulator of osteogenesis.

Because *Pten* deletion was insufficient to drive robust OS onset, we tested if co-deleting *Rb1* and *Pten* could synergistically promote osteosarcoma formation. Co-deleting *Rb1* with *Pten* greatly accelerated lipoma formation and increased the incidence to 100%, but did not induce osteosarcoma. It is possible that we failed to observe osteosarcoma because the rapid enlargement of lipoma tumors required mice to be euthanized around 3–4 months of age. We also aged *Osx1-Cre; Rb* ^*fl/+*^; *Pten* ^*fl/fl*^ mice to determine of retaining a single copy of *Rb1* promoted osteosarcoma formation. However, these mice only induced osteosarcoma in a single mouse (1/7), and developed lipomas at a rate and size intermediate between *Osx1-Cre; Pten* ^*fl/fl*^ and *Osx1-Cre Rb* ^*fl/fl*^; *Pten* ^*fl/fl*^ mice. These findings suggest that *Osx1-Cre* expresses cre in certain fat cells, or adipocyte precursors, outside the marrow, and that co-deleting *Rb1* and *Pten* in these cells promotes their sustained proliferation. In contrast, *Rb1* heterozygous co-deletion with *Pten* did not affect marrow adiposity or *Pten* driven bone outgrowth in the calvaria or femur trabeculation. Thus, mesenchymal cell fate outcomes, in regards to lipoma- vs osteosarcoma-genesis appear to depend heterozygous vs. homozygous gene deletion, the timing of gene loss during mesenchymal cell specification, and the capacity of the target cell to trans- or de-differentiate into other mesenchymal lineages.

## Supporting Information

S1 Fig Osteosarcoma presentation and tumor histology. A. Representative presentation of OS on the sternum of 12 month-old (A) *Osx-Cre; Pten* ^*fl/fl*^ and (B) *Osx-Cre; Rb1* ^*fl/+*^; *Pten* ^*fl/fl*^ mice. C. Tumor histology of OS from *Osx-Cre; Pten* ^*fl/fl*^ mouse. Bar = 500 μm. D. Tumor histology of OS from *Osx-Cre; Rb1* ^*fl/+*^; *Pten* ^*fl/fl*^ mouse. Bar = 500 μm. E & G. Histology of OS from *Osx-Cre; Pten* ^*fl/fl*^ mouse. Bar = 50 μm. Arrow notes adipocytes within the OS. F. Histology of OS from *Osx-Cre; Rb1* ^*fl/+*^; *Pten* ^*fl/fl*^ mouse. Bar = 50 μm. Arrow notes adipocytes within the OS. H. Osteoid-rich section of the tumor. Bar = 50 μm.
(PDF)

S1 Table A list of primers used in this study. (PDF)
