# Supplementary material for: Rb1 and Pten Co-Deletion in Osteoblast Precursor Cells Causes Rapid Lipoma Formation in Mice
Source: PLoS One. 2015 Aug 28;10(8):e0136729. doi: 10.1371/journal.pone.0136729 (PMC4552947; doi:10.1371/journal.pone.0136729)
Supplement: S1 Fig — A. Representative presentation of OS on the sternum of 12 month-old (A) Osx-Cre; Pten fl/fl and (B) Osx-Cre; Rb1 fl/+; Pten fl/fl mice. C. Tumor histology of OS from Osx-Cre; Pten fl/fl mouse. Bar = 500 μm. D. Tumor histology of OS from Osx-Cre; Rb1 fl/+; Pten fl/fl mouse. Bar = 500 μm. E & G. Histology of OS from Osx-Cre; Pten fl/fl mouse. Bar = 50 μm. Arrow notes adipocytes within the OS. F. Histology of OS from Osx-Cre; Rb1 fl/+; Pten fl/fl mouse. Bar = 50 μm. Arrow notes adipocytes within the OS. H. Osteoid-rich section of the tumor. Bar = 50 μm. (PDF) [file pone.0136729.s001.pdf]

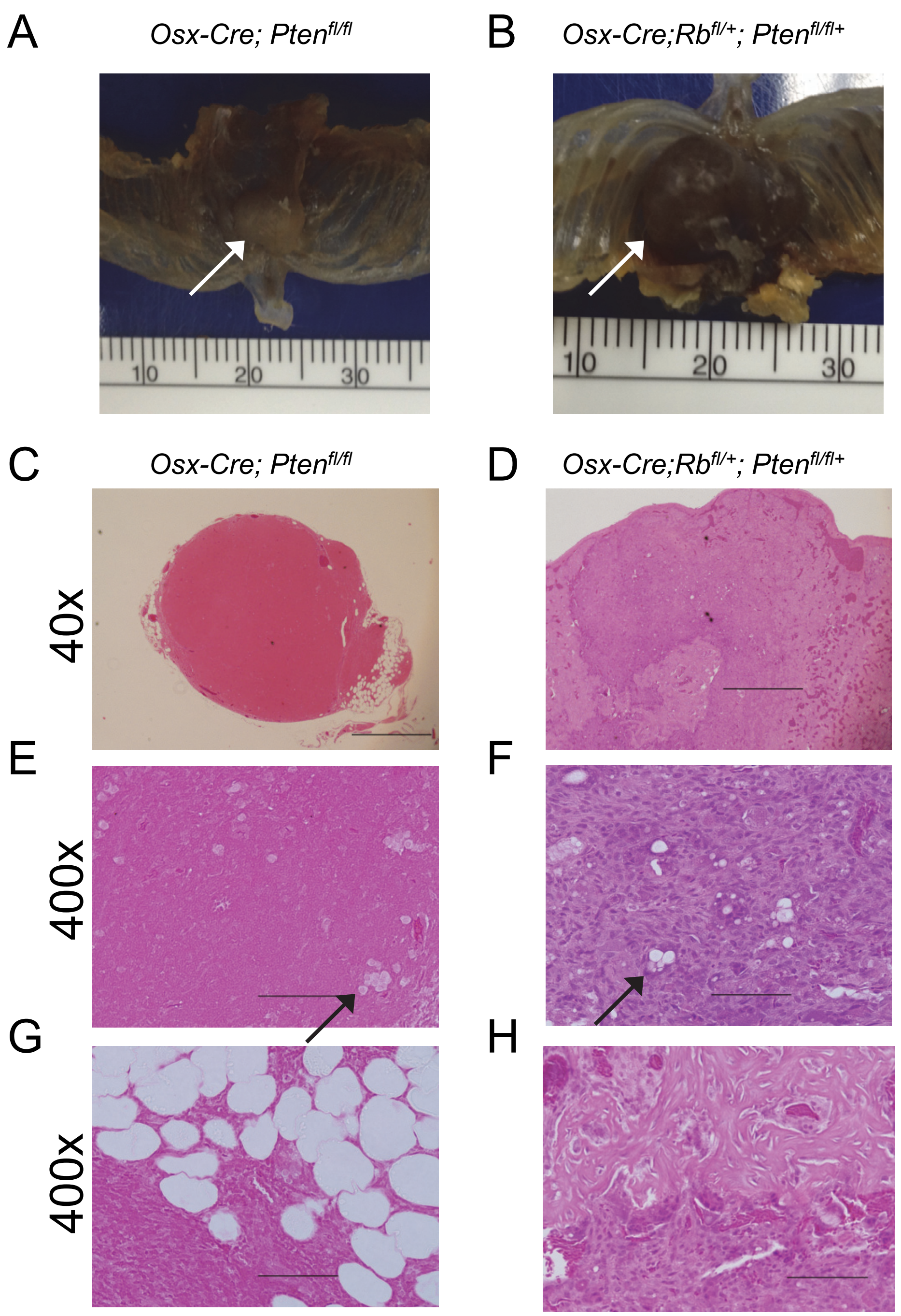

S1 Fig. Osteosarcoma presentation and tumor histology. A. Representative presentation of OS on the sternum of 12 month-old **(Figure A)** *Osx-Cre; Pten*$^{fl/fl}$ and **(Figure B)** *Osx-Cre; Rb1*$^{fl/+}$*; Pten*$^{fl/fl}$ mice. **(Figure C)**. Tumor histology of OS from *Osx-Cre; Pten*$^{fl/fl}$ mouse. Bar = 500 µm. **(Figure D)**. Tumor histology of OS from *Osx-Cre; Rb1*$^{fl/+}$*; Pten*$^{fl/fl}$ mouse. Bar = 500 µm. **(Figure E)** & **(Figure G)**. Histology of OS from *Osx-Cre; Pten*$^{fl/fl}$ mouse. Bar = 50 µm. Arrow notes adipocytes within the OS. **(Figure F)**. Histology of OS from *Osx-Cre; Rb1*$^{fl/+}$*; Pten*$^{fl/fl}$ mouse. Bar = 50 µm. Arrow notes adipocytes within the OS. **(Figure H)**. Osteoid-rich section of the tumor. Bar = 50 µm.
