# Supplementary material for: Rb1 and Pten Co-Deletion in Osteoblast Precursor Cells Causes Rapid Lipoma Formation in Mice
Source: PLoS One. 2015 Aug 28;10(8):e0136729. doi: 10.1371/journal.pone.0136729 (PMC4552947; doi:10.1371/journal.pone.0136729)
Supplement: S1 Table — (PDF) [file pone.0136729.s002.pdf]

Gene	Forward	Reverse
Alp (alkaline phosphatase)	TCTCCAGACCCTGCAACCTC	CATCCTGAGCAGACCTGGTC
Col1a1	CGAGTCACACCGGAACTTGG	GCAGGCAGGGCCAATGTCTA
Bglap (osteocalcin)	CTCTGTCTCTCTGACCTCACAG	CAGGTCCTAAATAGTGATACCG
Runx2	TGAGATTTGTGGGCCGGA	TCTGTGCCTTCTTGGTTCCC
Fabp4 (Ap2)	ATCCCTTTGTGGGAACCTGGAA	ACGCTGATGATCATGTTGGGCT
Cebpa	CAAGAACAGCAACGAGTACCG	GTCACTGGTCAACTCCAGCAC
Pparg	GAGCTGACCCAATGGTTGCTG	GCTTCAATCGGATGGTTCTTC
Ppargc1a (Pgc-1)	GTCCTCACAGAGACACTGGA	TGGTTCTGAGTGCTAAGACC

S1 Table. A list of primers used in this study.
